# Southern Branch of the Provincial Medical and Surgical Association

**Published:** 1836-10

**Authors:** 


SOUTHERN BRANCH OF THE PROVINCIAL MEDICAL AND SURGICAL ASSOCIATION.

We are happy to state that the Provincial Physicians and Surgeons resident in the Counties of Hants., Sussex, Wilts., Dorset, &c. are forming themselves into a local Association, subordinate to and strictly a part of the great original Provincial Association. Our readers are aware that a Branch Association has been already formed in the Eastern counties; and we doubt not that, before many years, the whole of the littoral counties of England will be portioned out into similar sectional and subordinate Associations. The effect of such unions cannot but be beneficial to the members and to the profession at large; and, so long as they are framed in strict accordance with the principles of the original Association, and are indeed merely local sections of it, they will themselves flourish and advance the interests of their parent; but no longer. That the southern branch of the Provincial Medical Association will be of this character, is evident from the terms of the circular calling the Preliminary Meeting for the purpose of establishing it, as in this the Requisitionists "solicit the aid of their brethren in the district for the purpose of forming an Association of Medical and Surgical Practitioners which may be considered as a branch or section of the Parent Society, to co-operate in its views and designs, to act in accordance with the spirit of its regulations, and to collect and embody such materials as may be deemed worthy the consideration of the great Association at its Annual Meetings."

